# Efficacy of a synuclein and tau fibril targeting drug in vivo

**DOI:** 10.1002/alz70859_106249

**Published:** 2025-12-25

**Authors:** Gregory M Cole, Cansheng Zhu, Kapil Manglani, Ke Hou, Alex Bombino, Mychica Jones, David S. Eisenberg, Sally A. Frautschy

**Affiliations:** ^1^ Veterans Greater Los Angeles Healthcare System, Los Angeles, CA USA; ^2^ Mary S. Easton Center for Alzheimer’s Disease Research at UCLA, Los Angeles, CA USA; ^3^ University of California Los Angeles, Los Angeles, CA USA; ^4^ Veteran Administration Geriatric Education Clinical Center, Los Angeles, CA USA; ^5^ UCLA, Los Angeles, CA USA; ^6^ University of California, Los Angeles, Los Angeles, CA USA; ^7^ Veterans Administration Geriatric Education and Clinical Center, Los Angeles, CA USA; ^8^ Howard Hughes Medical Institute, Los Angeles, CA USA

## Abstract

**Background:**

Pre‐Fibrillar oligomeric and insoluble fibrillar aggregates of alpha‐synuclein (aSyn) accumulate and contribute to the neurodegenerative decline in Parkinson Disease (PD) and other synucleinopathies and frequently occur as co‐morbidities in the major tauopathy, Alzheimer Disease (AD). Familial autosomal dominant PD aSyn A53T mutations which promote aggregation cause age‐related aSyn and tau pre‐fibrillar oligomers and insoluble aSyn fibrillar deposits, neurodegeneration and motor deficits in hemizygous A53T aSyn transgenic mice.

**Method:**

To test a candidate fibril structure‐based therapeutic candidate we treated aSyn deposit‐bearing 24 month old heterozygous A53T M83 mice for 6 weeks with a formulation of CNS11g, a small molecule designed to specifically fit an aSyn fibril site required for aggregation and previously demonstrated to disaggregate both pre‐existing aSyn and tau fibrils in cell free systems.

**Result:**

Oral gavage produced brain levels above the in vitro ED50 for disaggregation and ameliorated motor deficits with no evidence of toxicity compared with the vehicle group. CNS11g reduced levels of putatively neurotoxic, SDS‐stable, high molecular weight soluble aSyn aggregates detected above 256kD by Western blot analysis in spinal cord and brainstem as well as tau oligomers in spinal cord. Quantitative ICC for p129S aSyn deposits and reactive glia, supported a significant treatment effect, but there was no effect on detergent insoluble p129S aSyn. Our late intervention results provide evidence for effective oral CNS11g delivery, safety and efficacy in reducing motor deficits and soluble p129S aSyn and tau oligomers and aSyn deposits by ICC without biochemical evidence for reducing pre‐existing insoluble fibrillar aSyn deposits with this treatment paradigm.

**Conclusion:**

While higher or longer dosing might disaggregate and clear insoluble fibrils, this initial study suggests oral dosing produces pleiotropic CNS activity against both aSyn and tau pre‐fibrillar oligomers implicated in the neurotoxicity, seeding and spreading of two major proteinopathies.

